# The dual rod system of amphibians supports colour discrimination at the absolute visual threshold

**DOI:** 10.1098/rstb.2016.0066

**Published:** 2017-04-05

**Authors:** Carola A. M. Yovanovich, Sanna M. Koskela, Noora Nevala, Sergei L. Kondrashev, Almut Kelber, Kristian Donner

**Affiliations:** 1 Department of Biology, Lund University, Sölvegatan 35, 22362 Lund, Sweden; 2 Department of Biosciences, University of Helsinki, PO Box 65 (Viikinkaari 1), 00014 Helsinki, Finland; 3 A.V. Zhirmunsky Institute of Marine Biology, Far Eastern Branch of the Russian Academy of Sciences, ul. Palchevskogo 17, 690041 Vladivostok, Russia

**Keywords:** *Bufo*, *Rana*, photoreceptors, behaviour, visual threshold, colour vision

## Abstract

The presence of two spectrally different kinds of rod photoreceptors in amphibians has been hypothesized to enable purely rod-based colour vision at very low light levels. The hypothesis has never been properly tested, so we performed three behavioural experiments at different light intensities with toads (*Bufo*) and frogs (*Rana*) to determine the thresholds for colour discrimination. The thresholds of toads were different in mate choice and prey-catching tasks, suggesting that the differential sensitivities of different spectral cone types as well as task-specific factors set limits for the use of colour in these behavioural contexts. In neither task was there any indication of rod-based colour discrimination. By contrast, frogs performing phototactic jumping were able to distinguish blue from green light down to the absolute visual threshold, where vision relies only on rod signals. The remarkable sensitivity of this mechanism comparing signals from the two spectrally different rod types approaches theoretical limits set by photon fluctuations and intrinsic noise. Together, the results indicate that different pathways are involved in processing colour cues depending on the ecological relevance of this information for each task.

This article is part of the themed issue ‘Vision in dim light’.

## 1. Overview

The colour vision abilities of amphibians have been an intriguing subject for many decades, and it has repeatedly been hypothesized that these animals might be able to see colours at light intensities in which others can barely see anything. This idea stems from the presence of two spectrally different types of rods in most anurans (frogs and toads) and some urodeles (salamanders and newts) [1–3], first described by Franz Boll in 1877 [4] on the basis of their colour when viewed end-on in freshly dissected retinas. He used the term ‘red rods’ for the majority type found also in other vertebrates, and ‘green rods’ for the minority type that he found only in amphibian retinas. In 1955, Denton & Wyllie [5] showed that the absorbance of the ‘green rods’ peaks in the blue part of the spectrum at approximately 430 nm, whereas what they called ‘pink rods’ are typical vertebrate rhodopsin rods with absorbance maximum in the green part of the spectrum at approximately 500 nm. The traditional nomenclature is hopelessly confusing, so here we use the terms blue-sensitive (BS) rods and green-sensitive (GS) rods. The presence of photoreceptors that have different spectral sensitivities and are functional at the same light levels is mandatory for colour vision (see [6] for a review), and the fact that rods are active in dim light, when cones do not contribute to vision, led Denton and Wyllie to suggest ‘that frogs could have dichromatic colour vision using only their retinal rods’ [5].

In the last 50 years, a fair amount of information has accumulated about photoreceptor complements, opsin classes and signal processing in amphibian retinas. Table 1 summarizes the most relevant knowledge about the rod and cone complements of the two families of anurans in this study: BS rods, GS rods and BS, GS and red-sensitive (RS) cones.^1^

**Table 1.** Properties of the photoreceptors found in the retina of the most studied anurans from families Bufonidae and Ranidae. *Bb*, *Bufo bufo*; *Bg*, *Bufo gargarizans*; *Rp*, *Rhinella poeppigii* (formerly *Bufo marinus*); *Lp*, *Lithobates* (formerly *Rana*) *pipiens*; *Lc*, *Lithobates catesbeianus* (formerly *Rana catesbeiana*); *Rt*, *Rana temporaria*; n.d., no data available to our knowledge.

	BS rod	GS rod	BS cone	GS cone	RS cone
maximum absorbance or sensitivity (nm)	*Bb*: 432 [7]	*Bb*: 502 [7]	*Bb*: n.d.	*Bb*: n.d.	*Bb*: 562 [8]
	*Bg*: 432^a^	*Bg*: 502^a^	*Bg*: n.d.	*Bg*: n.d.	*Bg*: 562^a^
	*Rp*: 432 [7]	*Rp*: 503 [7]	*Rp*: n.d.	Rp: n.d.	*Rp*: n.d.
	*Lp*: 433 [9]	*Lp*: 503 [7]	*Lp*: n.d.	Lp: 502 [9]	*Lp*: 562 [10]
	*Lc*: 432 [7]	*Lc*: 502 [7]	*Lc*: 433 [11]	*Lc*: 502 [11]	*Lc*: 570 [11]
	*Rt*: 434 [7]	*Rt*: 503 [7]	*Rt*: 431 [10]	*Rt*: n.d.	*Rt*: 562 [10]
opsin	*Lc*: SWS2 [12]	all spp: Rh1 [13]	*Lc*: SWS1 [14]	n.d.	all spp: LWS [13]

^a^SL Kondrashev 2015, unpublished data.

The BS rods are thought to be ‘transmuted’ cones, evolutionarily modified to extend the operation of an ancestral cone receptor into a lower illumination range [15,16]. Accordingly, they possess *cone* pigments: in *Lithobates catesbeianus* BS rods have SWS2 while BS cones have SWS1 [12,14], whereas the latter pigment is found in both BS rods and cones in the urodele *Ambystoma tigrinum* [17]. Moreover, frog BS-rod pigment shows the fast regeneration after bleaching characteristic of cone pigments [18]. The rod-like morphology will in itself increase quantum catch and slow down responses (increasing temporal summation), but the transmutation also involves the use of rod instead of cone transducin, at least in *Ambystoma* [17]. There are no direct electrophysiological recordings from dark-adapted BS rods or cones of the species used in this study, however, and we must tentatively rely on results from other amphibians. The amplitude and kinetics of the single-quantum response of BS rods in the cane toad *Rhinella poeppigii* are very similar to those of GS rods [19,20]. The same is true of BS rods in salamander, where BS cones are 30 times less sensitive, in terms of photons impinging on the retina, due both to lower quantum catch and smaller single-quantum response [17]; yet, their dark-adapted response kinetics differ little. Comparing dark-adapted BS and RS cones, the former have four to five times higher gain, are much less noisy and have slower response kinetics [21]. All these differences suggest a higher sensitivity in the ‘blue’ than in the ‘red’ cone channel.

Despite all knowledge about amphibian retinal physiology, the hypothesis of rod-based colour vision in amphibians has never been strictly tested by behavioural experiments [22], and it is still unknown which photoreceptors are involved in colour vision at different light levels. The main obstacle for tackling these questions is the similarity in spectral sensitivities and response kinetics of BS rods and cones, which make their contributions virtually impossible to separate at light intensities where both rods and cones are active. Furthermore, rod intrusion in cone-dominated colour vision has been suggested for a number of species at mesopic light levels (reviewed by Kelber *et al*. [23]), so testing purely rod-based colour vision requires a firm knowledge of the limits of cone-based colour vision in these species.

The critical question is: can amphibians see colours at light intensities so low that significant cone contributions can be excluded based on their lower sensitivity? Thus, our objective in this study was to determine the lowest light levels where amphibians can discriminate colours. For the experiments, we relied on three behaviours: mate choice, prey-catching and phototaxis, using in all cases ‘blue’ and ‘green’ stimuli designed to stimulate GS and BS rods quantifiably and differently. The experiments were set up and adjusted at light levels where it is well known that the tested species can use colour cues (see [6] for a review and references) and then performed at a number of lower light intensities until a threshold level was found. Light intensity was expressed in two manners: (i) as (calculated) photoisomerization rates in rods, which allows us to relate performance to absolute limits and (ii) as luminance levels (cd m^−2^), which allows us to translate the experimental conditions into natural light scenarios and assess the ecological meaning. The spectral sensitivity curves for each photoreceptor and methods for calculating light intensities are detailed in the electronic supplementary material, parts S1 and S6.

In the three following sections, we provide the background, rationale and specific goals for each experiment along with experimental procedures and results. In the last section, we discuss the view of amphibian colour vision abilities that emerges from our present results together with previous evidence.

## 2. Mate choice experiments

In many anuran species, the breeding season lasts for just a few weeks of the year, during which the animals succumb to their sexual motivation. In George Orwell's words, ‘All he knows, at least if he is a male toad, is that he wants to get his arms round something, and if you offer him a stick, or even your finger, he will cling to it with surprising strength and take a long time to discover that it is not a female toad’ [24]. Such motivation has been fruitful for studying colour preferences of male frogs and toads by presenting them two or more ‘female models’ simultaneously, thus forcing them to decide which one to approach.

Previous research has shown that males of the common European frog (*Rana temporaria*) prefer red-coloured female models in their natural environment, but frogs are unsuitable for experiments in controlled illumination conditions as they lose sexual motivation when removed from the breeding pond [25–27]. On the other hand, breeding male toads of the genus *Bufo* display their characteristic sexual behaviour even in the laboratory, allowing for more detailed and controlled experiments. Such studies have shown species-specific differences in the colour preferences: *Bufo viridis* prefers black female models, whereas *B. gargarizans* and *B. bufo* prefer blue models and ignore those in the yellow-red range [25].

The sensitivity of male toads to the spectral composition of female models together with their willingness to make several choices in a row makes this experimental approach very well suited to test under which illumination conditions each of the amphibian colour channels (blue, green and red) works. With this strategy, we assessed the light intensities at which the differential stimulation of different colour channels stops contributing to mate choice behaviour.

### (a) Animals

Ten breeding couples of Asiatic toads *B. gargarizans* (formerly *B. bufo gargarizans*) were captured at Popov Island (Peter the Great Bay, Sea of Japan) during their migration from the forest where they hibernate to the breeding pond and transported to the laboratory. The experiments lasted 9 days; we used only the males and released all the animals in their natural environment afterwards.

Between the daily experimental sessions, the toads were kept in a dark room at 5–8°C in plastic vessels with wet soil, each vessel housing one breeding pair (male and female in amplexus). Before each experimental session, the toads were transferred to another vessel with a small amount of water and were adapted for 1 h at 20–22°C and luminance 2–9 cd m^−2^.

### (b) Colour stimuli and experimental design

**Figure 1.**
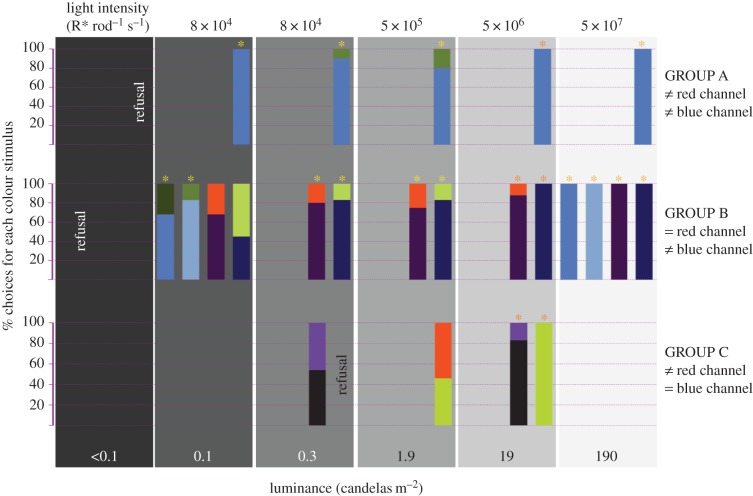
Proportions of choices of male toads for colour stimuli at different light intensities in mate choice experiments. Asterisks indicate significant preference for one of the colour stimuli in that pair and luminance level. The legend ‘refusal’ shows the cases in which the stimulus pair in that position was presented to the animals and failed to elicit the mating behaviour. The colour coding is only for guidance (the colours do not imitate those of the stimuli). The full dataset and statistics are available in the electronic supplementary material, part S3.

The set of stimuli was designed to dissect the contributions of the different colour channels in the amphibian retina (i.e. blue, green and red) at different light intensities, irrespective of the identity of the photoreceptors underlying them. We used blue and green as mentioned before, and also a few other colours to gather specific information about the dynamic range of the RS channel. The colour stimuli were paired to generate different excitation rates for each of the colour channels (see the electronic supplementary material, part S2), and the pairs were grouped on the basis of the relative excitation rates for the red and blue channels. In group A, the blue stimulus generates a higher signal in the blue, and lower in the red channel than its green counterpart. In group B, the blue/purple stimuli generate a higher signal in the blue channel than their green/orange counterparts, while the excitation in the red channel is virtually equal for both members of each pair. In group C, both components of each pair generate the same excitation for the blue channel while the signal in the red channel is higher for the purple/orange models than for their grey/green counterparts (see figure 1 for a summary of these grouping criteria). For all stimulus pairs that cause differential excitation of the green compared with the red channel that difference goes in the same direction but is smaller than the differential excitation of the blue channel. This led us to simplify the grouping and analyses by excluding green channel excitation as an independent variable.

We used the experimental procedure described in Gniubkin *et al*. [28]. The arena was a rectangle with 20 cm high walls covered with matte white paper. The female models used as stimuli consisted of stationary paper discs (3 cm diameter; [26]) printed in the selected colours and mounted on cardboard discs placed on the floor in front of one short end of the arena, equidistant from the edges and 30 cm apart from each other. The starting position for the animals in each trial was 50–70 cm from the stimuli. The arena was illuminated with a stabilized halogen source (24 v, 150 W) that reflected from a flat screen covered with Whatman filter paper to provide diffuse illumination and avoid shadows. The luminances used in these experiments were 190, 63, 19, 1.9, 0.3 and 0.1 cd m^−2^; they were achieved adding layers of neutral density glass filters (GOST USSR (State standard) 9411-75) in front of the light source.

In each experimental session, a couple of toads in amplexus were taken from the terrarium, the male carefully separated from the female and released in the arena. Before the two-choice trials, the male's motivation was tested with a single blue female model. Any male that did not approach it was excluded from the experimental session that day. We considered that the animal had made a choice when he approached one of the stimuli and grasped it with his forelegs. After that, the male was taken away from the arena and re-joined the female. The stimuli were changed and trials continued as long as the males maintained a steady motivation to make a choice (see the electronic supplementary material, video S1 for a demo of the experimental procedure in an open-air arena). As testing was constrained by the short time span of the breeding season and dependent on the motivation of the males, it was not possible to design a balanced experiment with a scheduled number of trials for each stimulus pair for each individual *a priori*. Thus, the choices made by all males for a given stimulus pair at each light intensity were pooled for the statistical analysis, adding up to more than 650 choices in total. The criterion for significant colour discrimination was the lower limit of the 95% confidence interval for proportions in binomial distributions [29] (see details in the electronic supplementary material, part S3).

### (c) Results

Our animals showed the behavioural pattern described for the species before, marked by an overall preference to approach the female models that generate a higher signal in the blue channel, and to avoid those that generate a higher signal in the red channel in bright light conditions. Figure 1 summarizes the results for all the stimulus pairs in all the tested light intensities. The behavioural choices in each of the groups show some clear patterns. In group A, the animals show the expected preference for the blue models in bright light but also at the previously untested lower light intensities. As in this pair the green stimulus compared with the blue stimulus produced not only less excitation of the blue channel, but also more excitation of the red channel, we cannot know which of these differences was most decisive at the different light levels. The results from group C show that when the only relevant difference in excitation rates happens in the RS channel, the discrimination of stimuli gets extinguished at luminance levels of 1.9 cd m^−2^ and lower. This result could indicate either that the photoreceptors underlying that channel are not sensitive enough at those light intensities, or that the aversive (red) stimulation becomes irrelevant. On the other hand, the results for group B show that when the stimuli differ mainly by the excitation of the BS channel they can be discriminated at lower light intensities, down to 0.1–0.3 cd m^−2^. This difference in the thresholds for the BS and RS channel supports the hypothesis of a higher sensitivity of the blue versus red cones that was mentioned in the first section.

The readiness and strong drive of the male toads for this innate response allowed us to test a large number of stimuli and showed that besides the colour preferences at higher light intensities, there is a range where the animals continue to grasp the female models even though they stop using chromatic cues. Still, the motivation of the males faded at light intensities several orders of magnitude higher than their absolute visual threshold [27]. Moreover, the known lack of sexual motivation of frog males in the lab made this behaviour unsuitable for a comparison between frogs and toads that would have yielded a more general picture of the colour vision abilities of anuran amphibians. To overcome these limitations, we turned to a ‘trainable’ behaviour.

## 3. Prey-catching experiments

Most adult anurans are carnivorous and rely heavily on motion detection for hunting prey. Their feeding behaviour lends itself very well to behavioural experiments, as beautifully described in the classic paper by Lettvin *et al*. [30]: ‘[The frog] will leap to capture any object the size of an insect or worm, providing it moves like one. He can be fooled easily not only by a bit of dangled meat but by any moving small object.’ Snapping for prey dummies has previously been used for determining the absolute visual threshold of *B. bufo* [31] and for demonstrating colour vision in *B. bufo* and *B. viridis* [27]. Colour-linked food rewards have also been used to test colour vision thresholds in salamanders [32] and other vertebrates like geckos [33]. The feeding behaviour based on prey features is trainable as well as seasonally stable, which makes it a promising experimental paradigm for testing the colour vision abilities in both *Bufo* and *Rana*.

### (a) Animals

We collected common toads (*B. bufo*; *n* = 5) and common frogs (*R. temporaria*; *n* = 3) at Lund University's biological station in Skåne, Sweden. The animals were kept in glass terraria, which were wrapped in light brown paper, with free access to water and hiding places, and fed with crickets and mealworms three times a week. The photoperiod (12 L : 12 D) and temperature (20°C) were kept constant throughout the experiments.

### (b) Colour stimuli and experimental design

The set of green-blue stimulus pairs used in this experiment was specifically designed to control for brightness cues. Brightness was calculated as the quantum catches provided by each colour to the different photoreceptors (electronic supplementary material, part S4). As it was not feasible to find a single pair in which blue and green would yield both the same quantum catches and maximum excitation for all photoreceptors, we resorted to several combinations that covered all the possible brightness relationships for each of them. We accomplished this with three different greens and three different blues combined in five pairs (electronic supplementary material, part S4). We printed the prey dummies (0.5 × 1.5 cm) in each of the selected colours for the two-choice experiments.

The arena was a Plexiglas terrarium with a built-in Y-maze wrapped in the same paper as the housing terraria. In each trial, there was one stimulus pair, with one prey dummy placed on each arm of the maze. Live mealworms placed in hidden compartments underneath each of the stimuli were used as rewards. The arena was inside a dark room and illuminated by a fluorescent tube (Phillips MASTER TL5 HO 90 De Luxe 24 W/950) at 1 m above the floor of the setup. Luminance levels of 40, 0.2, 0.004, 0.0004 and 0.00007 cd m^−2^ were achieved by adding layers of neutral density filters (Lee filters, Hampshire, UK) underneath the light source.

In each trial, both stimuli were moved simultaneously approximately 3 cm backwards and forward to elicit the prey-hunting behaviour. The first pilot trials showed an innate preference in the choice rate for the green prey, so we set that one as the ‘correct’ choice. The decision to snap at the green stimulus was rewarded by providing access to the prey item, while the choice of the blue stimulus was unrewarded (electronic supplementary material, video S2). Each experimental session consisted—ideally—of 10 consecutive stimulus presentations; the stimulus pairs were presented twice inverting the position (left/right) of each colour. The sequences for the presentations were assigned pseudo-randomly [34], and were different for each session. Whenever an animal stopped cooperating before the 10th trial, the session was put on hold and resumed the next day. Each animal performed two to four sessions per week, depending on their cooperativeness. To increase the motivation to hunt the prey dummies, the rewards during the experiments were the only food the animals received during this period.

The initial training took place at a luminance level of 40 cd m^−2^ and each animal performed at least 40 trials. As in the mate choice experiments, the threshold for colour discrimination was set at the lower limit of the 95% confidence interval for proportions in binomial distributions [29] (27 choices of green out of 40 total choices). Each individual reaching this criterion passed on to the second phase, in which 40 choices by each animal were collected with the same reward schedule at lower light intensities.

### (c) Results

All the animals were attracted by the moving stimuli and showed the prey-catching behaviour since the first trial, and they readily detected and ate the prey item that was offered as a reward for each correct choice.

**Figure 2.**
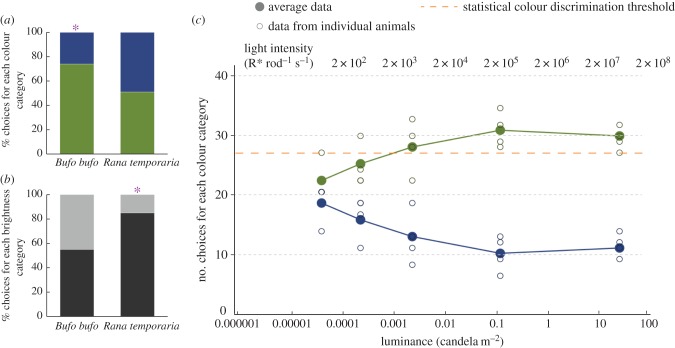
Prey-catching experiments results. The data from toads include only those animals that showed no significant side bias (four out of five). (*a*,*b*) Proportion of choices of toads and frogs for stimuli grouped by colour (*a*) and brightness (*b*) at 40 cd m^−2^. Asterisks indicate significant preference for one of the stimulus groups. (*c*) Performance of toads at different luminance levels. Stimuli are grouped by colour. See the electronic supplementary material, part S5 for full datasets and statistics.

After the initial 40 trials per individual, it was evident that our two species were choosing the preys in different ways. While the five toads were above the statistical threshold of 27 choices of green, the frogs' choice rates were very close to 50% for each colour. We did more trials with the frogs to give them the opportunity of putting aside whichever strategy they were using and ‘learn’ that they had to choose based on colour. After 120 trials, the choice rates were still statistically random regarding the colour of the prey dummies (figure 2*a*). When we sorted the choices by brightness of each stimulus instead of colour, a clear pattern emerged showing that frogs were mostly choosing the darkest available prey (figure 2*b*). The same analysis provided additional evidence that the toads' choices were actually driven by colour, as their responses sorted by brightness of the stimuli shows a random choice rate, indicating that the achromatic cue is irrelevant in their case. The choice patterns in both species were the same when the analysis was performed separately for each individual (see raw data in the electronic supplementary material, part S5) and stimulus pair (data not shown). Taken together, these results show that toads used colour vision in this experimental setting, as their choices were consistently ‘green’ irrespective of the brightness, whereas frogs used achromatic vision, as they consistently chose the stimuli with the lowest brightness regardless of its colour. As a consequence, frogs were excluded from the second phase. Before proceeding to the next stage, we sorted the toads' choices by position (left/right, data not shown) and confirmed that there was no side bias in four out of five animals. The only individual that showed a significant bias towards one side was excluded from the next stage, and his data are not included in figure 2.

The four remaining toads performed 40 trials each at each of the lower light intensities (figure 2*c*). Their choice rate for the green stimuli was above the statistical significance criterion for colour discrimination down to 0.004 cd m^−2^ for three out of four animals and 0.0004 cd m^−2^. The one animal that reached the statistical criterion at 0.00007 cd m^−2^ had failed in the previous step, so we consider that data point to be unreliable. While at 0.00007 cd m^−2^ the animals did not reach the significance criterion, all of them were still making choices and successfully spotting the prey item. These results show that the threshold for colour discrimination of toads in the prey-catching task is in the range 0.004–0.0004 cd m^−2^, while in the lowest part of the visual dynamic range the chromatic cue is not used. An equivalent experiment with human observers gave a colour threshold of 0.08–0.006 cd m^−2^ (data not shown). This result was expected considering the overall lower visual sensitivity of humans compared with amphibians, and is similar to previous findings [35].

Observing known differences in the optics of the eye and the dimensions, gains and integration times of the cones, dark-adapted cone vision in anurans at room temperature is estimated to be ≈100 times more sensitive than in humans [36,37]. Thus, the colour thresholds measured here may well be cone-determined and give no clear indication of rod involvement. Moreover, neither the mate choice nor the prey-catching experiment allowed determination of frog colour sensitivity. Therefore, we turned to the phototactic behaviour as our experimental paradigm for testing the performance of frog colour vision at low light levels.

## 4. Phototaxis experiments

Phototaxis, or the drive to orient and move in relation to a light source, is one of the simplest visual tasks that an animal can perform, as it only requires perception of the light direction [38]. Kühne (1878) [39] first observed that intact frogs moved from green towards blue light, while blinded individuals did not. In 1910, Pearse [40] summarized what was then known about amphibian colour preference: ‘The rays toward the violet end of the spectrum are apparently most potent in producing photic reactions, and the rays toward the opposite end approach in their effects the conditions brought about by dark.’ The question was approached again in classical studies on amphibian phototaxis and blue preference by Muntz [41,42] and Hailman & Jaeger [43,44], but in none of these was the question of absolute intensity thresholds addressed, either for the behaviour as such, or for the blue preference. In the 1980s, Aho *et al*. [45,46] developed a semi-automated high-throughput set-up to determine the absolute visual sensitivity of *R. temporaria* and *Lithobates pipiens*, taking advantage of the strong drive of the frogs to jump towards a light source when confined in a dark environment. Here, we adapted this set-up for the study of colour discrimination down to the absolute visual threshold. The purpose was threefold: (i) to pin down unambiguously the contributions of the two types of *rods* to colour vision, (ii) to get data from frogs, and (iii) to elucidate task-specific motivation issues in the previous experiments.

### (a) Animals

We used *R. temporaria* collected in the wild in southern Finland (seven females and 10 males). The animals were kept in basins with access to water at 16°C on a 12 L : 12 D photoperiod and force-fed with chicken liver and nutritious fish food after every experimental session. The basins were covered so that the frogs received only dim light. The experiments were performed during the light period (06.00 h–18.00 h), but the animals were kept in total darkness for at least 2 h before testing. The testing room temperature was kept constant at 18°C.

### (b) Colour stimuli and experimental design

**Figure 3.**
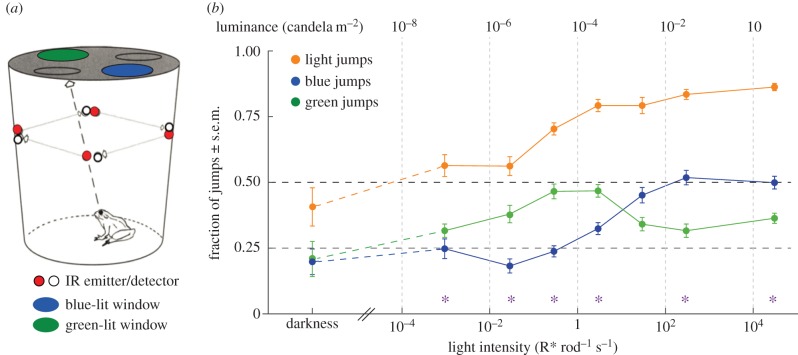
Phototaxis experiments. (*a*) Schematic drawing of a testing chamber. Adapted from Aho *et al*. [45]. (*b*) Fraction of jumps (mean ± s.e.m. of the fractions calculated for each frog separately) to each of the windows (blue jumps, green jumps) and to both lit windows together (light jumps = green + blue) as function of photoisomerization rates in GS rods. The green and the blue window are equivalent for GS rods and differ only by the additional stimulation of BS rods by the blue window. Measured luminances (cd m^−2^) in each experimental condition are shown on the upper abscissa for reference. Asterisks above the *x*-axis mark the light intensities where the total number of jumps towards the blue and the green window pooled across all sessions were distributed significantly differently from random 1 : 1 on a *χ*^2^-test. See the electronic supplementary material, part S8 for detailed datasets and statistics.

In these experiments, the stimuli were not reflecting objects, but two differently coloured lit windows (7 cm diam.) in diagonally opposite quadrants in the ceiling of a testing chamber (black plastic bucket; figure 3*a*). The two remaining quadrants were not open and are therefore referred to as ‘dark windows’. The only experimental variable was the intensity of the light homogeneously illuminating the entire arena (i.e. common to all windows), which is expressed as photoisomerizations per rod per second (R* rod^−1^ s^−1^) elicited over the retinal images of the lit windows (see the electronic supplementary material, part S6). The window colours were produced with Kodak Wratten 2 optical filters (no. 98, ‘blue’ and no. 8, ‘green’; Eastman Kodak Company, USA). The relative transmittances of the two windows were separately adjusted with neutral density filters in such a way that the ‘blue’ and ‘green’ windows stimulated GS rods equally. Given the spectral characteristics of the colour filters, photoisomerization rates in BS rods from the ‘blue’ window were then slightly (approx. 30%) higher than in GS rods, while BS rod stimulation from the ‘green’ window was about 20-fold lower (see the electronic supplementary material, part S7). For practical purposes, this is close enough to our original simple goal that BS/GS rod stimulation be ≈1 for the blue window and ≈0 for the green window. Although we keep these minor deviations in mind, we generally use the photoisomerization rate in GS rods as our only measure when considering the results (figure 3*b*).

The entire arena consisted of four testing chambers placed in a square array homogeneously lit from above by a common light. The primary light source was a 30 W halogen lamp driven by a stabilized current source (GWInstek GPS-3030 run at 2.3 A), enclosed in a light-tight box 112 cm above the roof of the arena and centred on the midpoint of the square array. The light passed through an edge filter cutting off wavelengths above 550 nm, neutral density filters to set the overall light intensity, and an acryl diffuser. In each of the four test chambers, four infrared emitter-detector pairs recorded the jumps into each of the quadrants (figure 3*a*). In each experimental session, four frogs were tested in parallel, one in each bucket. Each session was limited to last 1 h and each frog was tested only once per day. Each frog was tested four to eight times in each bucket and at least four times at each light intensity, adding up to a total of more than 20 000 recorded jumps.

### (c) Results

Figure 3*b* displays the fractions of jumps towards the blue and green window as function of light intensity. The orange curve (‘light’ jumps) is the sum of the blue and green jump fractions; ‘dark’ jump fractions (not shown to avoid clutter) are the complement of the orange curve. Random jumping would produce the fraction 0.5 of light jumps, distributed equally on blue and green (i.e. fraction 0.25 for each). These random levels are indicated by dashed lines in figure 3*b*. In total darkness (light source turned off), the fractions did not deviate statistically significantly from random, indicating that there was no inherent bias. To our surprise, however, a slight but significant rise of the green fraction was evident even from the lowest light intensity tested (0.001 R* rod^−1^ s^−1^; *χ*^2^-test of the distribution of jump numbers: *p* < 0.001; see the electronic supplementary material, part S8 for details about statistics). Given that the absolute threshold for seeing light at the same temperature reported by Aho *et al*. [45] is 0.01 R* rod^−1^ s^−1^ (albeit based on a stricter threshold criterion), this leads to the remarkable conclusion that frogs can discriminate colours as soon as they start seeing anything. At the next higher intensity tested here (0.02 R* rod^−1^ s^−1^), the green-blue difference becomes quite substantial, as green jumps increase while blue jumps drop significantly below chance level. Thus, ‘blueness’, i.e. a mere increase in the isomerization rate in BS rods, in fact acts as an *aversive* signal, making the blue window less attractive even than the dark quadrants. The aversive effect of BS rod stimulation at low intensities has the further paradoxical effect that apparent discrimination of ‘light’ (green + blue) from ‘darkness’ at this intensity stays close to chance level (1 : 1) even when there is very significant colour discrimination. From 0.2 R* rod^−1^ s^−1^ upwards blue jumps start increasing in parallel with green, but not until around 10 R* rod^−1^ s^−1^ upwards does blue become more attractive than green, as expected on the basis of previous studies [41–44]. This is already a range where the BS cones are active, and the relative role of the BS rods is uncertain. Consistent with this, it is also where human subjects (*n* = 3) viewing the same stimuli at the same distance as the frogs first reported seeing ‘blue’.

This remarkable sensitivity of colour discrimination lies near the physical limits set by the quantum character of light, as can be seen from the following estimations of signal-to-noise ratios (SNR) in our experimental conditions. The retinal image of the window covers about 30 000 GS rods and 3000 BS rods [47]. Over this area, the light intensity 0.001 R* rod^−1^ s^−1^ (where green and blue are already distinguished) produces a total of around 30 R* s^−1^ in GS rods and 4 R* s^−1^ in BS rods. Assuming 3 s integration time at this temperature [45,46,48], the *signal* for discrimination of blue from green is 3 × 4 = 12 R* and the *noise* (Poisson standard deviation of quantal fluctuations) is √(90 + 12) ≈ 10 R*. The SNR based on the photon flux alone is then SNR_in_ = 1.2 (cf. [46]).

This is by definition an upper limit. A more realistic measure of discriminability requires that intrinsic neural noise liable to obscure the signal be taken into account to give a physiological signal-to-noise ratio (SNR_out_). The most inexorable noise source is the random occurrence of spontaneous thermal activations of visual pigment molecules causing electrical ‘dark events’ in the rod cells that cannot even in principle be distinguished from responses to single photons. Dark event rates have never been directly measured in *R. temporaria* rods and extrapolation from other sources is unusually difficult in this case. Reported dark event rates in BS rods of the classical amphibian model, the toad *R. poeppigii* (*Bufo marinus*), span two orders of magnitude (0.0003 versus 0.06 R* rod^−1^ s^−1^) [19,20]. For GS rods, the situation is not much better: estimates in different species for 502-nm rod pigments with A1 chromophore range from 0.02 (toad) to 0.001 (salamander) R* rod^−1^ s^−1^ [49]. The lower estimates would enable high efficiency in the discrimination task (SNR_out_ remarkably close to SNR_in_), whereas the higher estimates would be associated with serious loss of reliability.

As a cautious solution, we may fall back on whole-retina dark-noise estimates from ganglion-cell recordings in *R. temporaria*, which translate into an equivalent rod event rate of 0.017 R* rod^−1^ s^−1^ at the temperature of the present experiments [45]. This would depress SNR_out_ at our discrimination threshold to around 0.3. Such low values are not generally considered useful in human detection tasks, but as the number of trials (jumps) is ‘unlimited’ here, it is enough to produce a significant bias.

## 5. Discussion

### (a) The use of colour by amphibians depends on context and light levels

The results underscore how the use of colour as a visual cue works differently for different behaviours and in different illumination ranges (see fig. 3 from Kelber *et al*. [23] for comparison with other animals and approximate luminances of natural light environments). These observations remind us to be cautious in generalizing sensory thresholds from a particular behaviour, as limitations may have more to do with the relevance of the specific cue and its interaction with other sensory signals in a given situation than with fundamental physical and physiological mechanisms. Another example known since the 1950s is that toads, as opposed to frogs, do not use colour cues for the optomotor response, even when the chromatic contrast is perfectly visible to them in other behaviours [28,50,51].

The behavioural thresholds provide some hints about the ecological relevance of colour. In the mate choice experiments, the male toads used colour for choosing the female models down to 0.3 cd m^−2^, which is the approximate luminance of a clear evening after sunset. Even if this species is primarily nocturnal or crepuscular, the diel pattern for breeding is flexible, and some studies even suggest that *B. bufo* prefers to mate under full moon rather than moonless nights [52]. A similar reasoning can be applied to the prey-catching behaviour. The luminance threshold for colour vision in this behaviour, around 10^−4^ cd m^−2^, is equivalent to a moonless, clear, starlit night. Still darker environments (e.g. prey-catching on a cloudy moonless night or under a thick canopy at night) need not be dealt with very often. Moreover, even if colour was a relevant cue for prey-catching in nature, it is certainly dispensable: toads will happily go on trying to catch prey in achromatic mode even at such low light intensities that the slowness of rod responses to the very sparse photon fluxes severely degrades the accuracy of hitting moving targets [31,48].

The situation is quite different for the phototactic behaviour. The scenario of being inside a dark enclosure is totally realistic and probably a frequent occurrence in nature. Finding an exit is of vital importance and would be expected to draw on all available information, including colour. Blue preference has been demonstrated in tens of frog and toad species [43,44] at photopic light levels, and it may be speculatively related to the blueness of the sky. In the same vein, our seemingly paradoxical finding that the wavelength preference is reversed at the very lowest light levels might make sense, as the primary nocturnal light sources—the stars and the moon—have comparatively reddish spectra [53]. Thus, phototactic orientation towards light of longer wavelengths might be purposeful on a dark night when only rods are active, whereas the blueness of the sky even at twilight is bright enough to activate BS cones. It is intriguing to think that signals from the spectrally near-identical BS rods and cones are, at some level of the visual system, wired for opposite phototactic responses to ‘blueness’.

### (b) Photoreceptor mechanisms underlying colour discrimination in the different tasks

A major goal of this study was to analyse the possibility of amphibian colour discrimination being based on signals from the two types of rods (BS and GS). In the mate choice behaviour, the colour discrimination threshold lay within the photopic range (even for the less sensitive human cone system), and at lower light levels the choices relied on achromatic cues and suggest no involvement of BS rods.

In the prey-catching experiments, the colour discrimination threshold was certainly lower than in humans, but can still be accounted for without rod involvement, as amphibian cones and especially BS cones are remarkably sensitive [17,21,37]. Thus, the most parsimonious interpretation of the prey-catching results would be that colour vision and its threshold are determined by the BS cones. Interestingly, in the mate choice experiments the threshold of the RS channel was found to be higher than that of the BS channel, consistent with the lower sensitivity and higher noise of RS cones [21].

### (c) Neural mechanisms of blueness discrimination

‘Blueness’ signals in anuran retinal ganglion cells and their brain projections have been studied over several decades (e.g. [8,42,54–57]). However, all these studies have been performed at photopic light levels at a time before BS cones were discovered [10,11], and thus the blue inputs were automatically attributed to BS rods. The fact is that nothing is known about the connectivity of BS rods. The observation that GS and BS signals have opposite behavioural effects allows no conclusions about the neural level where the opponency is established. The extreme blue-sensitivity of the phototactic response, where just a few photoisomerizations in BS rods when added to a 10 times higher rate in GS rods triggers aversive behaviour, suggests that it could rely on comparison of signals transmitted by parallel pathways up close to the motor output. If we trust recent, exceptionally low estimates of dark event rates in BS rods [19], the possibility of a privileged line from these to the brain appears especially intriguing.

In the more general context of visual strategies, it comes as a surprise that behaviour reflects opponency of signals from spectrally different rods even at the absolute visual threshold. According to common wisdom, all photon signals should then be pooled to maximize absolute sensitivity. However, investment in parallel pathways does make it possible to ‘eat the cake and have it too’, i.e. get around the trade-off between sensitivity (pooling all signals) and resolution (splitting and comparing signals in the spatio-temporal or, as here, the chromatic domain).

The frog retina is known to contain rare types of ganglion cells [27] not accommodated in the classification by Lettvin *et al*. [30]. For example, there is a type that sums ‘blue’ responses over receptive fields covering large parts of the retina [57] (studied at photopic levels). The question of neural circuits for colour signalling in amphibians really requires renewed investigation, including an overhaul of ganglion-cell classification with the battery of state-of-the-art methods that has been applied to mouse retina in the last few years (e.g. [58]). As a result of that work, at least 30 functional classes of mouse ganglion cells are now distinguished, making the paradigmatic Lettvin complement of amphibian ganglion-cell classes seem poor by comparison.

### (d) Concluding remarks

The data presented here show the lowest intensity threshold for colour discrimination in any animal species studied so far [23], supporting the long-standing hypothesis of rod-based colour discrimination in amphibians and highlighting the importance of finding behavioural tasks that are relevant for the animals in the experimental conditions in which they are tested [27]. On the other hand, the threshold values obtained in the different experiments show how a battery of different behaviours can unveil the existence of different pathways for processing colour information. Combining this kind of approach with electrophysiological studies will undoubtedly be useful to elucidate opponency mechanisms, connectivity of retinal networks and dimensionality of colour vision in different species.

## Supplementary Material

Detailed description of methods, analyses and original data

## Endnote

1 A detailed review of the current knowledge about visual systems of amphibians is beyond the scope of this article. However, it is worth mentioning that despite a handful of other species being fairly well studied (e.g. the South African clawed frog, *Xenopus laevis*, and a few salamanders/newts), ‘complete’ datasets about the most basic photoreceptor features are unavailable even for the most popular model species.

## Data accessibility

The datasets supporting this article have been uploaded as part of the electronic supplementary material.
