# Supplementary material for: The dual rod system of amphibians supports colour discrimination at the absolute visual threshold
Source: Philos Trans R Soc Lond B Biol Sci. 2017 Apr 5;372(1717):20160066. doi: 10.1098/rstb.2016.0066 (PMC5312016; doi:10.1098/rstb.2016.0066)
Supplement: Detailed description of methods, analyses and original data [file rstb20160066supp1.pdf]

Supplementary material for Yovanovich et al., 2017, The dual rod system of amphibians supports colour discrimination at the abso-lute visual threshold, *Phil. Trans. R. Soc. B.* doi: 10.1098/rstb.2016.0066

CONTENTS

1. SPECTRAL SENSITIVITY CURVES 1
2. STIMULI FOR MATE CHOICE EXPERIMENTS 2
3. COMPLETE DATASET FROM MATE CHOICE EXPERIMENTS 3
4. STIMULI FOR PREY CATCHING EXPERIMENTS 4
5. COMPLETE DATASET FROM PREY CATCHING EXPERIMENTS 6
6. CALCULATION OF ROD PHOTOISOMERIZATION RATES 7
7. STIMULI FOR PHOTOTAXIS EXPERIMENTS 8
8. COMPLETE DATASETS FROM PHOTOTAXIS EXPERIMENTS 9
9. REFERENCES 10

1. SPECTRAL SENSITIVITY CURVES

The absorbance spectra were calculated using the template for vitamin A1-based pigments and the absorbance maxima (for the blue- and green-sensitive rods of *Bufo bufo* and *Rana temporaria*) from Govardovskii and co-workers [1]. Correction for the self-screening effect in the long outer segments of rods was done according to the formula:

$$S_i = 1 - 10^{(-k*l*Abs)}$$

Where k is the absorption coefficient of the photoreceptor (1/µm), l is the length of the outer segment (µm) and Abs is the original absorbance spectrum obtained with the Govardovskii template. Please note that the absorbance spectrum, once corrected for self-screening effect, becomes the absorptance spectrum, and the latter is the one used throughout our work. To our knowledge, values of k and l have not been determined for some photoreceptors of *B. bufo* and *R. temporaria*; in those cases we used the values available from *Rhinella poeppigii* (formerly *Bufo marinus*) and *Lithobates* (formerly *Rana*) *pipiens* (Supplementary Table 1.1).

Photoreceptor	Parameter	Value	Species	Reference
Blue-sensitive rod, "*Bufo*"	Maximum absorbance	432	*Bufo bufo*	[1]
	Absorption coefficient (*k*)	0.032	*Rhinella poeppigii*	[2]
	Outer segment length (*l*)	33	*Rhinella poeppigii*	[3]
Green-sensitive rod, "*Bufo*"	Maximum absorbance	502	*Bufo bufo*	[1]
	Absorption coefficient (*k*)	0.039	*Rhinella poeppigii*	[2]
	Outer segment length (*l*)	45	*Bufo bufo*	[4]
Blue-sensitive rod, "*Rana*"	Maximum absorbance	434	*Rana temporaria*	[1]
	Absorption coefficient (*k*)	0.035	*"Standard vertebrate"*	[2]
	Outer segment length (*l*)	30	*Lithobates pipiens*	[5]
Green-sensitive rod, "*Rana*"	Maximum absorbance	503	*Rana temporaria*	[1]
	Absorption coefficient (*k*)	0.037	*Lithobates catesbeianus*	[6]
	Outer segment length (*l*)	43	*Rana temporaria*	[7]
Red-sensitive cone, "*Rana*"	Maximum absorbance	562	*Rana temporaria*	[8]
	Absorption coefficient (*k*)	Not used; self-screening negligible		
	Outer segment length (*l*)			

Supplementary Table 1.1. Values used for calculation of spectral sensitivity curves and self-screening correction of the photoreceptors in the species used throughout this study.

Regarding *Bufo gargarizans*, its absorbance maxima are the same than in *B. bufo* (see Table 1 in the article); so the uncorrected spectral sensitivities of both are exactly the same. There is no information available to our knowledge for estimating self-screening in *B. gargarizans*, but being very closely related to *B. bufo* we assumed that the effect would be the same so we used the self-screening corrected *B. bufo* curves for both species.

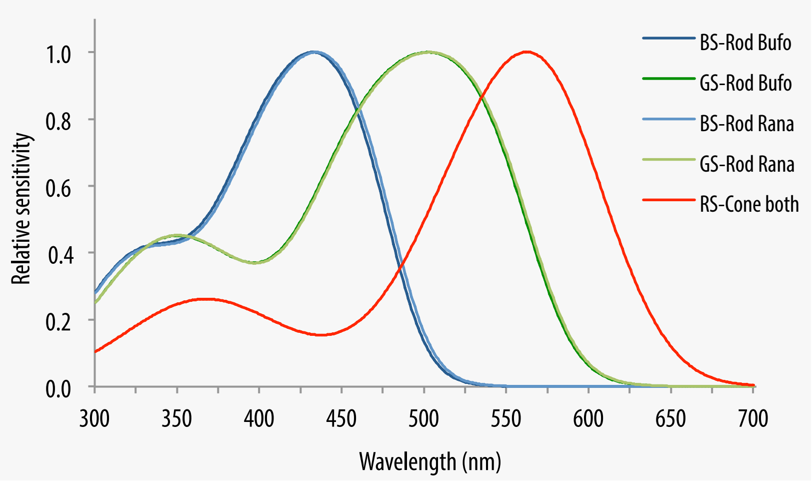

Supplementary Figure 1.1. Spectral sensitivity curves of the photoreceptors in the species used throughout this study.

For the red-sensitive cones we only had accurate data from *Rana temporaria* so we used that absorbance maximum for both of our model species. The outer segments of cones are very short so we considered the self-screening effect in their case to be negligible, as it has been done before [8]. The spectral sensitivity curves obtained here (Supplementary Figure 1.1) are used in the upcoming sections for calculations of quantum catches and photoisomerization rates.

2. STIMULI FOR MATE CHOICE EXPERIMENTS

The irradiance of the light source and the reflectances of the coloured papers used in these experiments (Supplementary Figures 2.1 and 2.2, respectively) were measured with an USB-2000+ spectrometer (Ocean Optics, Dunedin, FL, USA).

The reflectance curves were used to calculate the excitation that each coloured stimulus generated on each photoreceptor relative to the excitation of a Teflon white standard. Calculations were done using the following equation [9]:

$$R = \sum_{400\,nm}^{700\,nm} E(\lambda)r(\lambda)S(\lambda)$$

Where E is the irradiance of the light source, r is the reflectance of the stimulus, and S is the spectral sensitivity curve of the photoreceptor. The shape of the spectral sensitivity curve depends only on the absorbance maximum and the cromophore, and not on the photoreceptor type [1]. On the other hand, even though there is no specific information about the absorbance maxima of blue-sensitive cones in *Bufo*, the close similarities for all photoreceptor types among Bufonids and Ranids

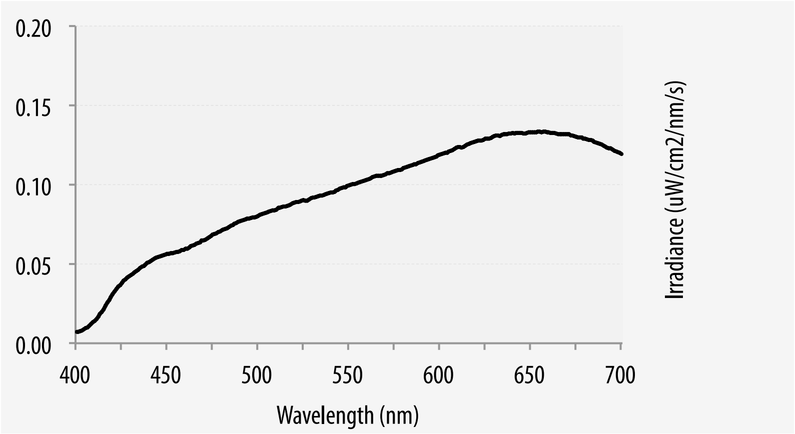

Supplementary Figure 2.1. Irradiance of the light source in mate choice experiments.

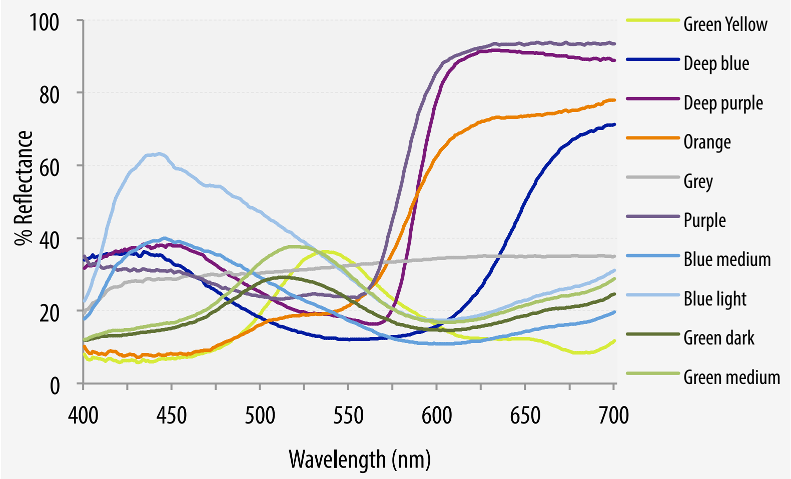

Supplementary Figure 2.2. Reflectance curves of the stimuli used in mate choice experiments. The colour coding is approximate and only for guidance.

(see Table 1 in the article) led us to assume that the spectral sensitivity curve for the blue-sensitive photoreceptors would be the same regardless of their cone or rod nature and to refer to them simply as "colour channels". The same reasoning was applied to green-sensitive photoreceptors.

Supplementary Figure 2.3 shows the relative excitation values obtained for each colour stimulus and channel.

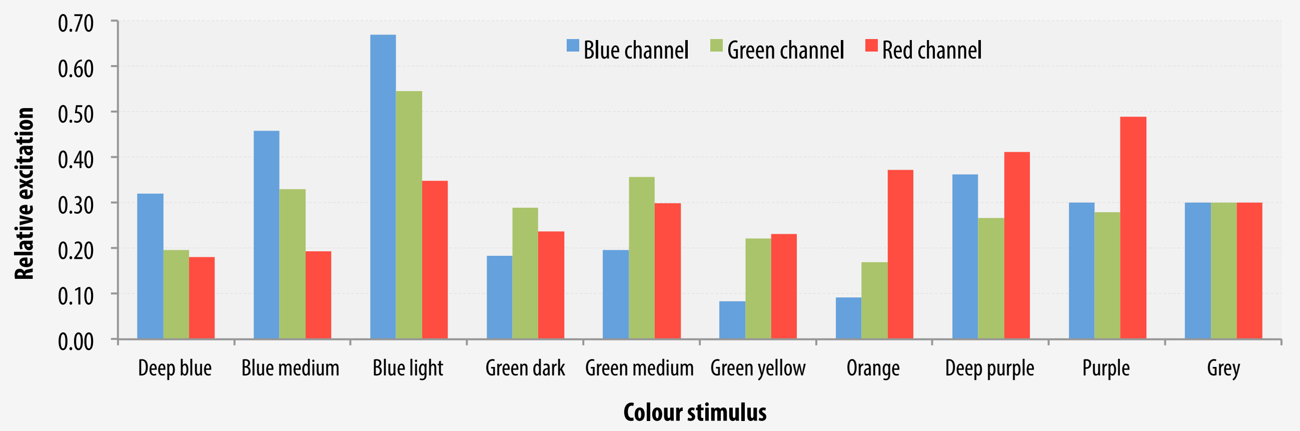

Supplementary Figure 2.3. Relative excitations of the different colour stimuli for each colour channel in the toads' retina.

To quantify the differences between the relative excitations provided by each colour stimulus to each channel we calculated the Michelson's contrast as follows:

$$MC_i = \frac{(R(x)_i - R(y)_i)}{(R(x)_i + R(y)_i)}$$

Where $R(x)$ and $R(y)$ are the relative excitations from each of the two colours being compared and i is the channel. The values for the selected stimuli pairs are shown in Supplementary Table 2.1.

Pair N°	Stimuli	BS-channel	GS-channel	RS-channel	Grouping
1	Green yellow (GY) vs. Deep blue (BB)	-0,589	0,060	0,121	B
2	Green yellow (GY) vs. Orange (OO)	-0,051	0,132	-0,234	C
3	Deep purple (DP) vs. Orange (OO)	0,595	0,222	0,050	B
4	Grey (GG) vs. Purple (PP)	0,000	0,034	-0,241	C
5	Green medium (GM) vs. Blue medium (BM)	-0,402	0,040	0,217	A
6	Green medium (GM) vs. Blue light (BL)	-0,547	-0,209	-0,075	B
7	Green dark (GD) vs. Blue medium (BM)	-0,429	-0,065	0,103	B

Supplementary Table 2.1. Michelson contrasts comparing the relative excitations of colour stimuli from the pairs selected for the mate choice experiments. Highlighted in grey are the values within the range considered as virtually equal excitation (±0.15). The last column shows the grouping of the pairs (according to the relative excitations for each colour channel) that was used to build Figure 1 in the article.

3. COMPLETE DATASET FROM MATE CHOICE EXPERIMENTS

Here we provide all the data from mate choice experiments and statistical analyses. The lower limit of the 95% confidence intervals for proportions based on binomial distributions were obtained from [10] and represent the minimum number of items in the sample that must belong to one of the two categories in order to consider that the difference between categories is statistically significant.

Pair N°		Luminance (cd/m²) 190	63	19	1,9	0,3	0,1	<0,1
1	Deep Blue	20	NOT TESTED	20	15	20	9	NOT TESTED
	Green Yellow	0		0	3	4	11	
	95% confidence limit	15		15	14	17	15	
	Stats	S		S	S	S	NS	
2	Green Yellow	REFUSED TO MAKE CHOICES	NOT TESTED	20	11	REFUSED TO MAKE CHOICES	NOT TESTED	NOT TESTED
	Orange			0	13			
	95% confidence limit			15	17			
	Stats			S	NS			
3	Deep Purple	20	NOT TESTED	23	42	16	23	NOT TESTED
	Orange	0		3	14	4	11	
	95% confidence limit	15		19	36	15	24	
	Stats	S		S	S	S	NS	
4	Grey	NOT TESTED	NOT TESTED	19	NOT TESTED	14	NOT TESTED	NOT TESTED
	Purple			4		12		
	95% confidence limit			19		19		
	Stats			S		NS		
5	Blue medium	20	NOT TESTED	20	16	18	20	REFUSED TO MAKE CHOICES
	Green medium	0		0	4	2	0	
	95% confidence limit	15		15	15	15	15	
	Stats	S		S	S	S	S	
6	Blue light	20	NOT TESTED	NOT TESTED	NOT TESTED	NOT TESTED	20	REFUSED TO MAKE CHOICES
	Green medium	0					4	
	95% confidence limit	15					17	
	Stats	S					S	
7	Blue medium	20	NOT TESTED	NOT TESTED	NOT TESTED	NOT TESTED	30	NOT TESTED
	Green dark	0					14	
	95% confidence limit	15					29	
	Stats	S					S	

Supplementary Table 3.1. Detailed results of mate choice experiments and statistical analyses. S: Significant; NS: Non significant.

4. STIMULI FOR PREY CATCHING EXPERIMENTS

For this experiments we generated a large set of blue and green samples to select the optimal stimuli to test blue-green discrimination. The irradiance of the light source and reflectances of the stimuli were measured with a Maya 2000 spectrometer (Ocean Optics, Dunedin, FL, USA). The curves for the lamp and the chosen colours are shown in Supplementary Figures 4.1 and 4.2, respectively.

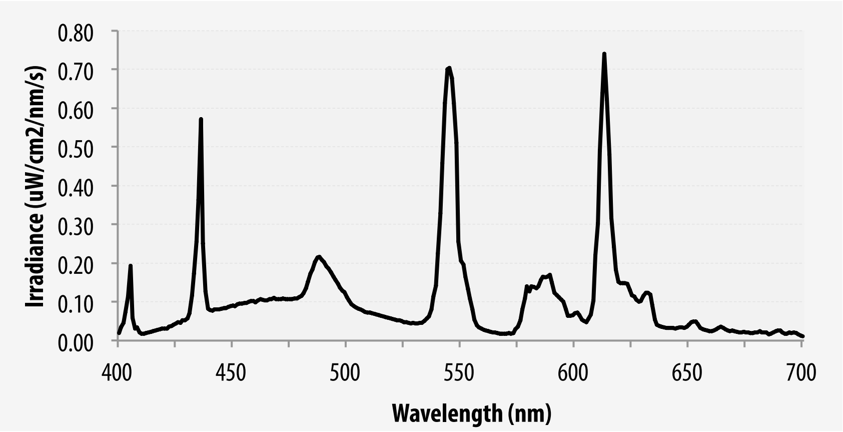

Supplementary Figure 4.1. Irradiance of the light source used in prey catching experiments.

To quantify how bright each colour stimulus looks for the photore-

ceptors, we measured the irradiance of the samples and the light source with a spectrometer and used those spectra in photons $(cm^2)^{-1}$ s^{-1} nm^{-1} to calculate the quantum catches (Q) of each photoreceptor "i" for each of the colours using the following equation [11]:

$$Q_i = \sum_{300nm}^{700nm} E(\lambda)S_i(\lambda)$$

Where $E(\lambda)$ is the irradiance spectrum in photon units and $S_i(\lambda)$ is the absorbance spectrum of the photoreceptor. Supplementary Table 4.1 shows the obtained Q values for all stimuli as well as the background paper used in the prey-catching experiments. Given the striking similarity of the spectral sensitivity curves of *Bufo* and *Rana* the Q values are virtually the same for both species. These data were used to decide how to combine the colours to form the stimuli pairs reported in the article.

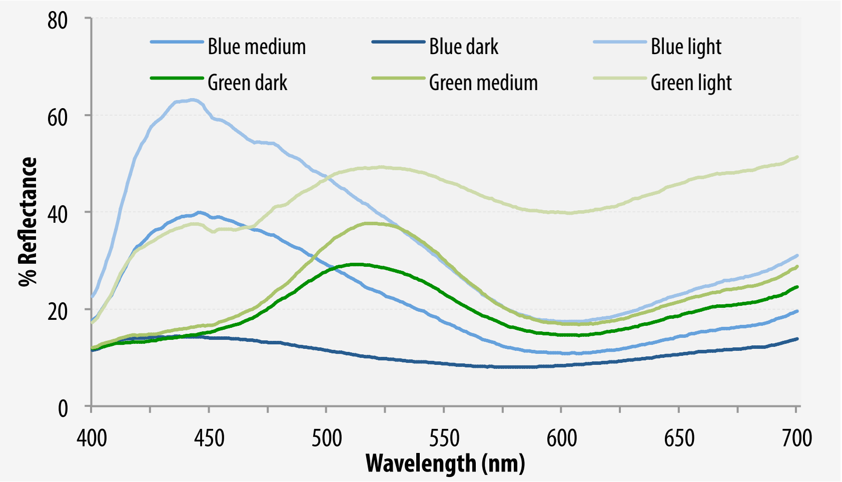

Supplementary Figure 4.2. Reflectance curves of the stimuli used in prey catching experiments. The colour coding is approximate and only for guidance.

Colour	Q catches (x10^{11})			Corresponding wavelength for the blue-green dichromatic system
	BS-Rod	GS-Rod	RS-Cone	
Blue light	105	193	184	473
Blue medium	68	115	105	472
Blue dark	29	47	55	471
Green light	61	187	276	483
Green medium	32	116	150	485
Green dark	28	92	116	484
Background paper	56	127	189	477

Supplementary Table 4.1. Quantum catches of the colour stimuli used in the prey catching experiments.

Besides fulfilling the brightness relationships that we wanted to test for each of the photoreceptors, in the prey catching experiment we aimed for all the "blues" to be as equally "blue" as possible, and the same with the "greens". To quantify that we calculated their corresponding wavelength [12] for the dichromatic blue-green sensitive rod system using the formula:

$$\text{Ratio} = Q_{GSrod} / (Q_{BSrod} + Q_{GSrod})$$

The resulting values showed in Supplementary Table 4.1 confirm that all the variants of blue and green are quite close to the rods' absorbance maxima and remarkably similar within each other -within a 3 nm range-, so it is safe to assume that they were perceived as different intensities of the same colour by the animals. The background paper was selected for having a perceived wavelength intermediate between our blue and green stimuli – such that none of the stimuli would blend into the background – and also quantum catch values that fell in the middle of the range. To quantify the differences in the quantum catches of the different colours for each photoreceptor we used the Michelson contrast as explained in part 2 of this document and selected the stimuli pairs shown in Supplementary Table 4.2.

Supplementary Table 4.2. Michelson contrasts comparing the quantum catches of colour stimuli from the pairs selected for the prey catching experiments. Highlighted in grey are the values within the range considered as equally bright (±0.05). This information was used to build Table 3 in the article.

Pair N°	Stimuli	BS-rod	GS-rod	RS-cone
1	Green medium (GM) vs. Blue medium (BM)	-0,367	0,000	0,178
2	Green dark (GD) vs. Blue dark (BD)	-0,016	0,315	0,353
3	Green light (GL) vs. Blue dark (BD)	0,351	0,594	0,667
4	Green dark (GD) vs. Blue light (BL)	-0,578	-0,357	-0,230
5	Green medium (GM) vs. Blue light (BL)	-0,540	-0,251	-0,102

In these experiments we aimed for a strict control of achromatic cues, so the colours were considered equally bright only when the contrast value was ≤0.05.

5. COMPLETE DATASET FROM PREY CATCHING EXPERIMENTS

The detailed dataset of colour choices for all *Bufo bufo* and *Rana temporaria* individuals at the brightest light intensity is shown in Supplementary Table 5.1.

Supplementary Table 5.1. Performances of toads and frogs in the prey catching experiment at 40 cd/m^2 sorted by stimulus colour for all stimuli pairs. Data from the toad Bruno were excluded after he showed a significant bias to the stimuli on the right side of the maze. This information was used to build Figure 2A in the article.

Species	Animal	Green choices	Blue choices	Total choices
Bufo bufo	Baltasar	29	11	40
	Bernardo	30	10	40
	Blas	27	13	40
	Braulio	32	8	40
	**Total *Bufo***	**118**	**42**	**160**
Rana temporaria	Ramón	57	63	120
	René	64	56	120
	Ricardo	64	56	120
	**Total *Rana***	**185**	**175**	**360**

To test for choice biases based on stimuli brightness we sorted the behavioural choices as “dark/bright” instead of “green/blue” for each stimuli pair. The brightness/darkness relationships for each pair of stimuli can change depending on which photoreceptor is looking at them, so in this part of the analysis we excluded data from pair 1, which has opposite brightness patterns for different photoreceptors (see part 4 of this document), not allowing to assign unequivocally the “bright/dark” categories. The sorted data is shown in Supplementary Table 5.2.

Supplementary Table 5.2. Performances of toads and frogs in the prey catching experiment at 40 cd/m^2 sorted by stimulus brightness for stimulus pairs 2, 3, 4 and 5. This information was used to build Figure 2B in the article.

Species	Animal	Dark choices	Bright choices	Total choices
Bufo bufo	Baltasar	17	15	32
	Bernardo	22	10	32
	Blas	16	16	32
	Braulio	16	16	32
	**Total *Bufo***	**71**	**57**	**128**
Rana temporaria	Ramón	81	15	96
	René	84	12	96
	Ricardo	81	15	96
	**Total *Rana***	**246**	**42**	**288**

The detailed dataset for prey-catching experiments performed with *Bufo bufo* in decreasing luminance levels is shown in Supplementary Table 5.3.

Supplementary Table 5.3. Total number of correct (i.e., green) choices out of 40 trials from individual toads in prey catching experiments at different luminance levels. The threshold value is 27 out of 40 (see Supplementary Material part 3). This information was used to build Figure 2C in the article.

Animal	Luminance (cd/m2)				
	**40**	**0,2**	**0,004**	**0,0004**	**0,00007**
Baltasar	29	28	22	24	20
Bernardo	30	29	30	22	20
Blas	27	32	28	22	27
Braulio	32	35	33	30	20
Average	30	31	28	25	22

6. CALCULATION OF ROD PHOTOISOMERIZATION RATES

The method for calculating the photoisomerizations rod^{-1} s^{-1} elicited by a given stimulus is not readily available in the literature, so here we provide a detailed step by step of the procedure we used:

1) Calculate the energy of single photons at different wavelengths

$$E = \frac{hc}{\lambda}$$

Where: h (Planck's constant) = (6.626 x 10^{-34} J s)

c (speed of light) = (299 792 458 m s^{-1})

2) Measure the stimulus power spectrum P (W cm^{-2} nm^{-1}); W=J s^{-1}

3) Combine 1 & 2 to calculate the photon flux spectrum that reaches the eye

$$F_{cornea} = \frac{P}{E} \qquad \left(= \frac{J}{cm^2 * nm * s} \Big/ \frac{J}{photon} = \frac{photons}{cm^2 * nm * s}\right)$$

4) Calculate how much of that reaches the retina, and the size of the retinal image on which the light falls

$$F_{retina} = \frac{F_{cornea} * A_{pupil}}{A_{retina}} * \tau_{media} * \tau_{cornea}$$

A_{pupil} = dark adapted pupil area
A_{retina} = calculated retinal projection area (*)
τ_{media} = transmittance through ocular media
τ_{cornea} =1-corneal reflectance

(*)

$$A_{retina} = \pi * \left(\frac{d_{retina}}{2}\right)^2$$

$$d_{retina} = \frac{r}{R} * D_{stim}$$

Where: r = posterior focal length

R = distance from the stimulus to the eye

D_{stim} = stimulus diameter

5) Transform the photon flux spectrum to a photoisomerization spectrum

$$Total\ R^*_{photoR} = F_{retina} * Absorptance\ spectrum\ (Govardovskii) * (1 - 10^{-OD_{retina}}) * \gamma$$

Where: OD_{retina} = Mean optical density of the isolated retina as a whole measured at the λ_{max} of rhodopsin

γ = quantum efficiency of photoactivation/bleaching

6) Integrate the area under the curve to obtain the absolute number of photoisomerizations

7) Divide by rod density to obtain the N° of photoisomerizations/individual rod

$$R^*\ photoR^{-1}s^{\ -1} = \frac{Total\ R^*{}_{photoR}}{photoR\ density}$$

(The assumption is that absorption by other than rhodopsin rods represents negligible fractions of the total absorption).

As it becomes evident from the equations above, the number of R* $rod^{-1}s^{-1}$ depends on the dimensions and geometry of the experimental arena and stimuli used. Thus, the values need to be calculated independently for every experimental situation and are not readily convertible into photometric units, even in cases in which the ambient illumination measured, for example, in cd/m^2 would be the same for different experiments. The values used for *Rana temporaria* in the phototaxis experiments are as follows:

$A_{pupil} = 10\ mm^2$ [13]

$\tau_{media} \approx 1$ [14]

$\tau_{cornea} = 0.91$ [15]

$r = 0.45$ cm [15]

$OD_{retina} = 0.34$ [16]

$\gamma = 0.66$ [17]

$R = 26$ cm

$D_{stim} = 7$ cm

$A_{retina} = 1.13\ mm^2$

7. STIMULI FOR PHOTOTAXIS EXPERIMENTS

The irradiance spectra of the light source and the light passing through the filters used in these experiments are shown in Supplementary Figure 7.1.

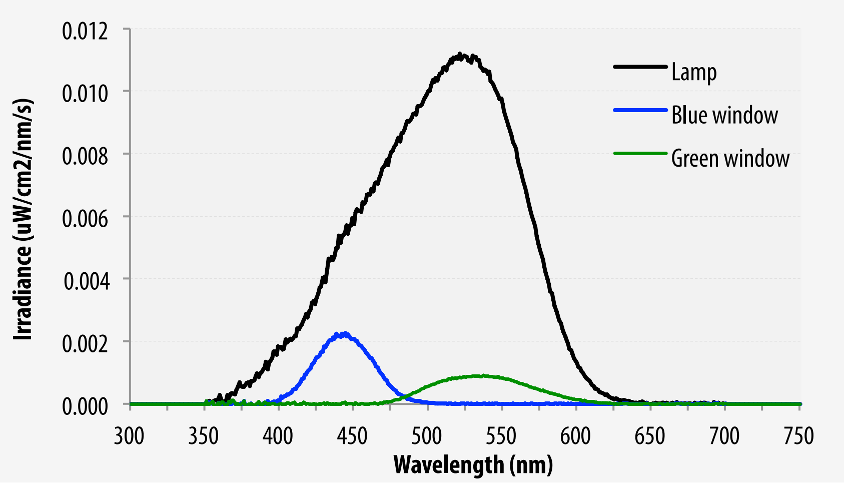

Supplementary Figure 7.1. Irradiance of the light source and filters used in phototaxis experiments.

The photoisomerization rates produced by the two windows (blue and green) in the two types of rods (BS and GS) are shown as stimulus spectra [R* $rod^{-1}\ s^{-1}\ nm^{-1}$] in Supplementary Figure 7.2. The total photoisomerization rate [R* $rod^{-1}\ s^{-1}$] in each case is obtained as the integral of the respective spectrum (the area under the curve). The light intensities of the two windows were independently adjusted with neutral density filters so that both produced approximately equal rates of photoisomerizations in GS-rods, as given in Supplementary Table 8.1. The rates in BS-rods are then inevitably fixed by the spectral characteristics of the colour filters (Wratten N°8, "green" and N° 98, "blue"; Eastman Kodak Company USA). The filters were basically selected with the aim that BS-rod stimulation by the green filter should be negligible, whereas GS- and BS-rod stimulation by the blue filter should be equal. This could not be perfectly achieved given the limited complement of Wratten filters available: rather than the ideal BS/GS-rod stimulation ratios of 0 (green) and 1 (blue), the actual ratios were about 0.05 (green) and 1.3 (blue). We think these deviations from the ideal are in practice negligible at the low light levels that are of main interest in this study of rod-based colour discrimination (≤ 1 R* $rod^{-1}\ s^{-1}$). It is worth noting that the smallest *achromatic* difference that a frog can detect at the lowest light levels (the increment threshold) is 100%, i.e., doubling the intensity of a very dim background light [13].

Supplementary Figure 7.2. Photoisomerization rates (R* rod^{-1} s^{-1} nm^{-1}) produced in GS- and BS-rods by the "green" and "blue" stimuli used in the phototaxis experiments, measured at the maximum light intensity. Adjustment of the general illumination with neutral density filters means that all spectra are scaled down by a common factor in each experimental condition. The area under each curve yields total R* rod^{-1} s^{-1}.

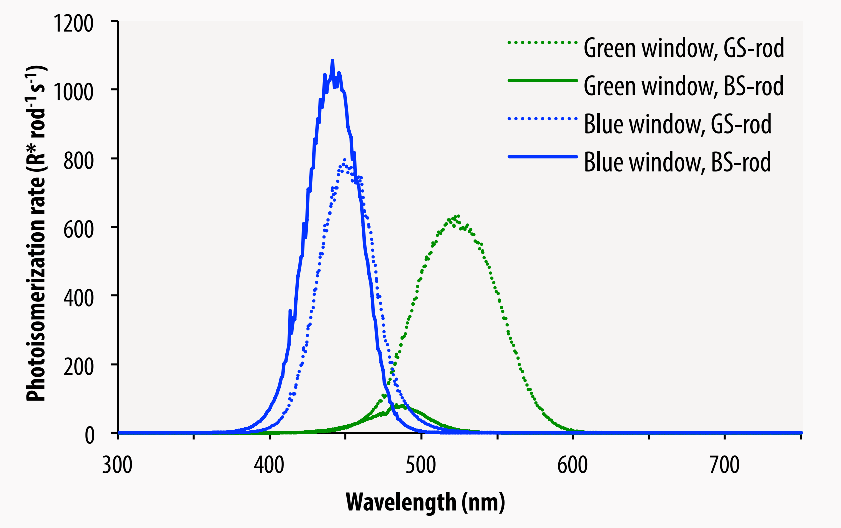

8. COMPLETE DATASETS FROM PHOTOTAXIS EXPERIMENTS

The output from the infrared jump-recording system was processed to yield the total jump counts shown in Supplementary Table 8.1. These data were used for statistical analysis of jump distributions by χ^2 testing: (1) jumps towards lit sectors (green + blue) vs. dark sectors; (2) jumps towards blue vs. green. In both cases the "expected" (random) distributions are 1:1.

Intensity (R* rod^{-1} s^{-1})	Animals	Blue jumps	Green jumps	Dark jumps	X^2 test (Light vs. Dark)	X^2 test (Blue vs. Green)
Darkness	15	163	193	355	p>0.05	p>0.05
0.001	15	247	404	536	p<0.001	p<0.001
0.03	17	359	732	911	p<0.001	p<0.001
0.3	17	528	995	958	p<0.001	p<0.001
3.01	17	1085	1453	915	p<0.001	p<0.001
30.12	17	1536	1485	898	p<0.001	p>0.05
301.18	17	1567	1250	698	p<0.001	p<0.001
30118.04	17	1896	1327	634	p<0.001	p<0.001

Supplementary Table 8.1. Raw data and statistics from phototaxis experiments at different light intensities. The p values marked in red indicate the cases in which the number of jumps towards each of the compared conditions was statistically significantly different.

To ease visualization of the data, the absolute jump counts of each frog were used to calculate the fractions of jumps made by that individual towards each of the sectors at each light intensity (number of jumps towards that sector divided by the total number of jumps of that individual at that intensity). Means ± SEMs of the fractions were calculated across all frogs for each light intensity; these values are shown in Supplementary Table 8.2 and displayed as data points and error bars in Fig. 3B.

Intensity (R^* rod^{-1} s^{-1})	Fraction Blue	Blue SEM	Fraction Green	Green SEM	Fraction Dark	Dark SEM	Fraction Light	Light SEM
Darkness	0.19813	0.0487	0.20866	0.06672	0.59322	0.07273	0.40678	0.07273
0.001	0.24774	0.03737	0.31622	0.02549	0.43604	0.04167	0.56396	0.04167
0.03	0.18229	0.0267	0.37968	0.03329	0.43803	0.03577	0.56197	0.03577
0.3	0.23771	0.02117	0.46574	0.02814	0.29655	0.02303	0.70345	0.02302
3.01	0.32445	0.02257	0.46793	0.02404	0.20762	0.0232	0.79238	0.0232
30.12	0.45132	0.02935	0.34107	0.02541	0.20761	0.03073	0.79239	0.03073
301.18	0.51833	0.02754	0.31623	0.02542	0.16544	0.01906	0.83456	0.01906
30118.04	0.49902	0.02411	0.36363	0.01933	0.13735	0.01367	0.86265	0.01367

Supplementary Table 8.2. Results of phototaxis experiments at different light intensities expressed as mean values across frogs of the fractions of jumps calculated separately for each frog, and standard errors of the means. This dataset was used to build Figure 3B in the article.

9. REFERENCES

[1] Govardovskii VI, Fyhrquist N, Reuter T, Kuzmin DG & Donner K. 2000 In search of the visual pigment template. *Visual neuroscience* **17**, 509-528.

[2] Warrant EJ & Nilsson DE. 1998 Absorption of white light in photoreceptors. *Vision Res* **38**, 195-207. (doi:10.1016/S0042-6989(97)00151-X).

[3] Harosi FI. 1975 Absorption spectra and linear dichroism of some amphibian photoreceptors. *The Journal of general physiology* **66**, 357-382.

[4] Aho AC, Donner K, Helenius S, Larsen LO & Reuter T. 1993 Visual performance of the toad (Bufo bufo) at low light levels: retinal ganglion cell responses and prey-catching accuracy. *J Comp Physiol A* **172**, 671-682.

[5] Nilsson SE. 1964 An Electron Microscopic Classification of the Retinal Receptors of the Leopard Frog (Rana Pipiens). *Journal of ultrastructure research* **10**, 390-416.

[6] Donner K, Firsov ML & Govardovskii VI. 1990 The frequency of isomerization-like 'dark' events in rhodopsin and porphyropsin rods of the bull-frog retina. *The Journal of physiology* **428**, 673-692.

[7] Hemila S & Reuter T. 1981 Longitudinal spread of adaptation in the rods of the frog's retina. *The Journal of physiology* **310**, 501-528.

[8] Koskelainen A, Hemila S & Donner K. 1994 Spectral sensitivities of short- and long-wavelength sensitive cone mechanisms in the frog retina. *Acta physiologica Scandinavica* **152**, 115-124. (doi:10.1111/j.1748-1716.1994.tb09790.x).

[9] Maximov VV, Orlov OY & Reuter T. 1985 Chromatic Properties of the Retinal Afferents in the Thalamus and the Tectum of the Frog (Rana-Temporaria). *Vision Res* **25**, 1037-1049. (doi:Doi 10.1016/0042-6989(85)90092-6).

[10] Rohlf FJ & Sokal RR. 1995 *Statistical tables*, Macmillan.

[11] Johnsen Sn. 2012 *The optics of life : a biologist's guide to light in nature*. Princeton, NJ, Princeton University Press; x, 336 p., 338 p. of plates p.

[12] Roth LS, Balkenius A & Kelber A. 2007 Colour perception in a dichromat. *The Journal of experimental biology* **210**, 2795-2800. (doi:10.1242/jeb.007377).

[13] Aho AC, Donner K & Reuter T. 1993 Retinal origins of the temperature effect on absolute visual sensitivity in frogs. *The Journal of physiology* **463**, 501-521.

[14] Govardovskii VI & Zueva LV. 1974 Spectral sensitivity of the frog eye in the ultraviolet and visible region. *Vision Res* **14**, 1317-1321.

[15] Aho AC, Donner K, Hyden C, Reuter T & Orlov OY. 1987 Retinal Noise, the Performance of Retinal Ganglion-Cells, and Visual Sensitivity in the Dark-Adapted Frog. *J Opt Soc Am A* **4**, 2321-2329. (doi:10.1364/Josaa.4.002321).

[16] Donner K, Koskelainen A, Djupsund K & Hemila S. 1995 Changes in retinal time scale under background light: observations on rods and ganglion cells in the frog retina. *Vision Res* **35**, 2255-2266.

[17] Dartnall HJ. 1968 The photosensitivities of visual pigments in the presence of hydroxylamine. *Vision Res* **8**, 339-358.

© 2017 The Authors. Under the terms of the Creative Commons Attribution License http://creativecommons.org/licenses/by/4.0/, which permits unrestricted use, provided the original author and source are credited.
